# Comparison of Herbal and Potassium Nitrate Toothpastes in Managing Dentin Hypersensitivity: A Randomized Controlled Trial

**DOI:** 10.3390/dj13080369

**Published:** 2025-08-15

**Authors:** La-ongthong Vajrabhaya, Supranee Benjasupattananan, Kraisorn Sappayatosok, Papatpong Sirikururat, Suwanna Korsuwannawong, Vittawin Dechosilpa

**Affiliations:** 1 College of Dental Medicine, Rangsit University, Pathumthani 12000, Thailand; la-ongthong.v@rsu.ac.th (L.-o.V.); supranee.b@rsu.ac.th (S.B.); kraisorn.s@rsu.ac.th (K.S.); papatpong.s@rsu.ac.th (P.S.); 2 Research Office, Faculty of Dentistry, Mahidol University, Bangkok 10400, Thailand; suwanna.aut@mahidol.ac.th

**Keywords:** herbal product, Java Tea, Little Ironweed, *Orthosiphon aristatus*, *Vernonia cinerea*, anti-sensitivity toothpaste, dentin hypersensitivity

## Abstract

**Background/Objectives**: This study evaluates the effectiveness of a toothpaste containing Java Tea and Little Ironweed in alleviating tooth sensitivity compared to a conventional potassium nitrate toothpaste. **Methods**: A total of 90 healthy patients aged 18–70 with up to two teeth exhibiting gingival recession were recruited into this study. All selected teeth had a visual analog scale (VAS) score ≥ 4 in response to tactile or air blast stimuli. Excluded teeth included those requiring restoration or participants undergoing treatments affecting sensitivity or taking pain medication/anti-sensitivity agents. Participants were randomly divided into three groups and instructed to brush twice daily with different toothpastes. The S1 group was prescribed toothpaste containing Java Tea and Little Ironweed extract, the S2 group was prescribed toothpaste containing Java Tea and Little Ironweed extract with 0.7% potassium nitrate, and the S3 group was prescribed toothpaste containing potassium nitrate. VAS scores were recorded at baseline, 2 weeks, and 4 weeks. Data were then compared and statistically analyzed between the groups. **Results**: For the tactile test, the final number of included teeth was 47 in the S1 group, 46 in the S2 group, and 22 in the S3 group. For the air blast test, the number of teeth included was 38 in the S1 group, 30 in the S2 group, and 27 in the S3 group. At baseline, mean VAS scores were comparable across groups for both tactile (S1: 6.89 ± 0.98; S2: 6.65 ± 1.52; S3: 6.82 ± 1.99) and air blast tests (S1: 7.39 ± 1.15; S2: 7.53 ± 1.31; S3: 6.89 ± 2.12). All groups showed significant reductions in VAS scores from baseline at both 2 and 4 weeks. A Kruskal–Wallis test indicated significant between-group differences in VAS scores at 2 and 4 weeks (*p* = 0.001). Post hoc analysis (Dunn’s test with Bonferroni correction) at 2 weeks revealed that the S1 group had significantly higher VAS scores than those of S2 (tactile *p* = 0.001, air blast *p* = 0.001) and S3 (tactile *p* = 0.002, air blast *p* = 0.018). By 4 weeks, the S2 group demonstrated superior efficacy, with significantly lower VAS scores compared to those of S1 (tactile *p* < 0.001, air blast *p* = 0.030) and S3 (tactile *p* = 0.035, air blast *p* = 0.001). **Conclusions**: All tested toothpastes effectively reduced dentin hypersensitivity over the study period. Potassium nitrate toothpaste provided more rapid initial relief, when compared to the herbal formulation alone; however, both achieved similar outcomes by 4 weeks. The herbal toothpaste supplemented with potassium nitrate demonstrated superior efficacy and may offer a promising natural alternative for managing dentin hypersensitivity.

## 1. Introduction

Dentin hypersensitivity is a common global condition, with a recent meta-analysis reporting that it affects more than one-third of the world’s population [1]. Dentin exposure, often resulting from gingival recession or the loss of enamel or cementum, increases the susceptibility of nerves to external stimuli [2]. Brännström (1963) proposed the hydrodynamic theory, which suggests that the movement of dentinal fluid in response to physical stimuli—such as pressure or temperature—activates baroreceptors on odontoblasts, thereby transmitting neural signals to the brain [3].

Various approaches exist to alleviate dentin hypersensitivity, including laser treatment [4], local application of desensitizing agents, and the prescription of anti-sensitivity mouthwashes and toothpastes [5]. Currently, two major categories of desensitizing toothpastes are available on the market. The first contains potassium salts, which function by interrupting the transmission of nerve impulses [6]. The second category includes toothpastes formulated with chemical agents that can occlude exposed dentinal tubules, thereby reducing dentin permeability and sensitivity [7].

The use of natural products in daily life, including in oral care, is gaining popularity worldwide. Several natural substances, such as *Spinacia oleracea* (spinach) [8], *Rheum rhabarbarum* (rhubarb) [9], and propolis [10,11], have demonstrated dentinal tubule-occluding properties and are considered as potential candidates for inclusion in desensitizing toothpaste formulations. In Southeast Asia, *Orthosiphon aristatus* (Java Tea) and *Vernonia cinerea* (Little Ironweed) are widely available herbs known to be rich in potassium salts [12,13], which may reduce the dentin sensitivity problem by depolarizing the nerve [14]. Furthermore, a recent in vitro study [15] found that a toothpaste containing Java Tea and Little Ironweed extracts occluded dentinal tubules. This effect was supported by both visual and quantitative evidence: scanning electron microscopy (SEM) showed fine, crystal-like potassium salt structures, and hydraulic conductance tests confirmed a 40% reduction in dentin permeability.

The first formula for these herbal toothpastes was launched in the market as a hybrid toothpaste containing Java Tea and Little Ironweed extract mixed with 0.7% synthetic potassium nitrate. Clinical studies have shown that this formulation significantly reduces dentin hypersensitivity, achieving effects comparable to those of conventional potassium nitrate-based toothpaste at both 2 and 4 weeks [16]. However, it remains unclear whether the potassium nitrate or herbal ingredients were responsible for the anti-sensitizing effects. Therefore, the present study aimed to evaluate the efficacy of a toothpaste formulated solely with Java Tea and Little Ironweed extracts in alleviating dentin hypersensitivity, in comparison with one of the same formula with the addition of potassium nitrate, and a conventional synthetic potassium nitrate toothpaste.

## 2. Materials and Methods

**Figure 1:**
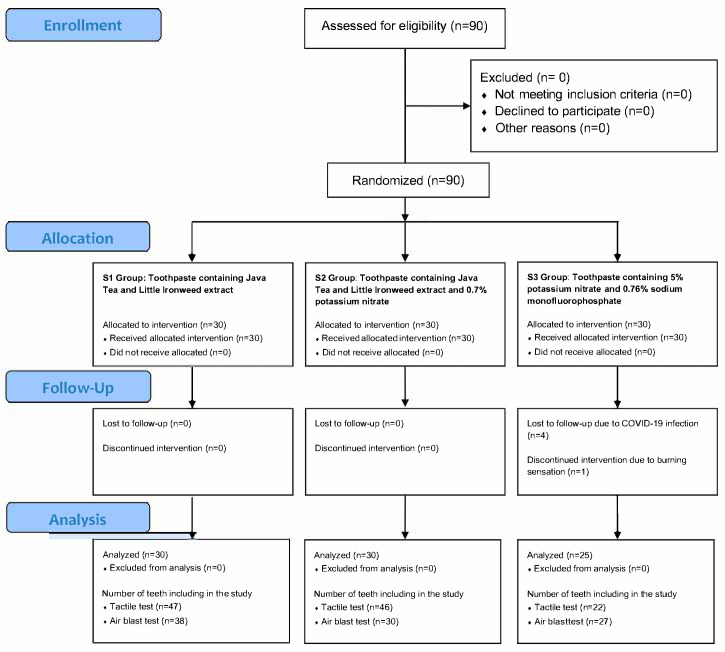
CONSORT RCT flow diagram.

This double-blind, randomized controlled trial was conducted at the College of Dental Medicine, Rangsit University between August 2020 and November 2022. The study protocol was approved by the institutional ethics committee, and all participants provided informed consent before enrollment. This study protocol was registered with the Thai Clinical Trail Registry (TCTR) on 16 June 2025. The TCTR identification number is TCTR20250616012, which can be found at https://www.thaiclinicaltrials.org/show/TCTR20250616012. The CONSORT RCT flow diagram was shown in Figure 1.

### 2.1. Eligibility Criteria

Participants were required to be in good general health and have no known allergies to Java Tea or Little Ironweed. Each participant needed to have at least 10 teeth remaining.

Eligible teeth met the following criteria:

- Exhibited gingival recession with exposed root dentin.
- Showed no evidence of dental caries or non-carious tooth surface loss requiring restorative intervention.
- Had a baseline Visual Analog Scale (VAS) score of ≥ 4 in response to either tactile or air blast stimulation.
- Presented no tooth mobility.

A maximum of two teeth per patient were selected for this study. If a patient had more than two eligible teeth, the tooth with the highest VAS score was chosen.

Exclusion criteria included the following:

- History of dental procedures that could influence dentin hypersensitivity within specific timeframes:
  ▪ Scaling and root planing within the past 30 days.
  ▪ Tooth whitening within the past 90 days.
  ▪ Orthodontic treatment within the past 90 days.
  ▪ Dental surgery involving the target tooth within the past 90 days.
- Use of painkillers, anti-sensitizing agents, or alternative anti-sensitizing toothpastes before and during the study.
- Pregnancy and breastfeeding.
- Any systemic health condition that might contraindicate participation in a clinical study (uncontrolled diabetes, a compromised immune system, etc.)

In the absence of previous research, the sample size was calculated through G*Power analysis (version 3.1). Based on data from a pilot study, 84 teeth across all groups were determined to be necessary to achieve 90% statistical power at a significance level of 0.05.

### 2.2. Sensitivity Assessment Protocol

VAS scores were recorded for each participant at the initial visit using a standardized protocol. The VAS scale ranged from 0 (no sensitivity) to 10 (intolerable sensitivity). For the tactile test, a dental explorer (EXD11-12 Hu-Friedy, Chicago, USA) was used to apply consistent pressure to the tooth surface for 10 s. The air blast test involved directing a blast of air from a triple syringe at 45–60 psi and a temperature of 17–21 °C onto the tooth surface at a distance of 10 mm for 1 s. To be included in the study, teeth had to exhibit a baseline VAS score of ≥4 in response to either tactile or air blast stimulation.

Following baseline measurements, 180 teeth from 90 participants aged 18–70 years were enrolled in the study. Each participant was randomly assigned by a computer-generated sequence to one of three groups. Participants were instructed to brush twice daily for 4 weeks with either the test toothpaste containing Java Tea and Little Ironweed extract (Twin Lotus Co. Ltd., Bangkok, Thailand/S1 group), toothpaste containing Java Tea and Little Ironweed extract with 0.7% potassium nitrate (Twin Lotus Co. Ltd., Bangkok, Thailand/S2 group), or the control toothpaste containing 5% potassium nitrate and 0.76% sodium monofluorophosphate (Sensodyne Fresh Mint^®^, GlaxoSmith Kline, London, UK/S3 group). The detailed composition of each toothpaste is presented in Table 1. To ensure blinding, all toothpastes were packaged in identical opaque containers labeled only with the group code (S1, S2, and S3). Both participants and researchers remained blinded to the composition until after the statistical analysis.

All participants received standardized oral hygiene instructions and were provided with a soft-bristle toothbrush and dental floss to maintain consistent oral care throughout the study. VAS score measurements were repeated at 2 and 4 weeks.

### 2.3. Statistical Analysis

Statistical analyses were conducted using SPSS version 22.0. As the data were not normally distributed, non-parametric tests were employed. A Friedman test was used to assess within-group differences in sensitivity over time. Post hoc analyses were conducted using a Bonferroni-corrected pairwise Wilcoxon signed-rank test for multiple comparisons. A Kruskal–Wallis test was used to compare VAS score reductions between groups at each time point. Post hoc analyses were conducted using Dunn’s test with a Bonferroni correction for multiple comparisons.

## 3. Results

During the study period, several participants were lost to follow-up, resulting in a reduced sample size in the S3 group. Notably, one participant in the S3 group experienced a burning sensation and subsequently withdrew from the study. For the tactile test, the final sample included 47 teeth in the S1 group, 46 in the S2 group, and 22 in the S3 group. Regarding the air blast test, the number of teeth included was 38 in the S1 group, 30 in the S2 group, and 27 in the S3 group.

### 3.1. Distribution of VAS Scores

At baseline, the VAS scores across all groups ranged approximately from 4 to 10 for both the tactile and air blast tests. After 2 weeks of toothpaste use, fewer than half of the evaluated teeth showed VAS scores below 4, which indicated a mild degree of sensitivity. Specifically, for the tactile test, 14.90% of teeth in the S1 group, 26.09% in the S2 group, and 45.46% in the S3 group achieved this reduction. For the air blast test, the respective percentages were 15.79% (S1), 26.67% (S2), and 25.92% (S3).

By the 4-week assessment, a greater proportion of teeth had VAS scores below 4, indicating increased effectiveness over time. For the tactile test, 59.58% of teeth in the S1 group, 78.36% in the S2 group, and 54.54% in the S3 group achieved this threshold. In the air blast test, these figures were 59.58% for the S1 group, 76.67% for the S2 group, and 40.74% for the S3 group. These results are summarized in Table 2 and Table 3.

### 3.2. Within-Group Comparison

VAS scores from both the tactile and air blast assessments revealed similar sensitivity reduction trends within all groups. The S1 group (Java Tea and Little Ironweed toothpaste) and S2 group (Java Tea, Little Ironweed, and potassium nitrate toothpaste) both showed significant reductions in sensitivity at the 2-week mark (S1: tactile *p* < 0.001, air blast *p* = 0.001, S2: tactile *p* = 0.001, air blast *p* < 0.001), with continued improvement at 4 weeks (S1: tactile *p* < 0.001, air blast *p* < 0.001, S2: tactile *p* < 0.001, air blast *p* = 0.002). In contrast, the S3 group (potassium nitrate toothpaste) exhibited a marked decrease in VAS scores at 2 weeks (tactile *p* = 0.002, air blast *p* = 0.005), but showed no statistically significant improvements between the 2- and 4-week assessments (tactile *p* = 0.366, air blast *p* = 0.341). These findings are detailed in Table 4 and Table 5.

### 3.3. Between-Group Comparison

At baseline, the mean VAS scores did not differ significantly between groups for either tactile (*p* = 0.392) or air blast (*p* = 0.306) stimuli, confirming comparable initial levels of dentin hypersensitivity. At the 2-week evaluation, both the S2 and S3 groups demonstrated significantly greater reductions in dentin hypersensitivity compared to those in the S1 group. This result was indicated by significantly lower VAS scores for S2 (tactile *p* < 0.001, air blast *p* < 0.001) and S3 (tactile *p* < 0.002, air blast *p* = 0.018) when compared to the score for S1. By the 4-week assessment, the S1 and S3 groups exhibited comparable reductions, suggesting similar long-term efficacy. Notably, the S2 group maintained a greater reduction in VAS scores at 4 weeks than the reductions observed in the S1 group (tactile *p* < 0.001, air blast *p* < 0.030) and S3 group (tactile *p* = 0.035, air blast *p* < 0.001). These results are presented in Table 6 and Table 7.

## 4. Discussion

This study investigated the desensitizing effects of a toothpaste containing extracts of Java Tea (*Orthosiphon aristatus*) and Little Ironweed (*Vernonia cinerea*), both of which are known to be rich in potassium salts [12,13]. These potassium compounds are believed to act similarly to potassium nitrate, the active ingredient in the control toothpaste.

A potassium nitrate-containing toothpaste was used as the control, as it possesses the ability to interrupt the nerve action potential. A study in 2017 further demonstrated that this compound is also capable of occluding the dentinal tubule, as shown in the SEM section [17]. These results suggest the potential of a dual-action mechanism for anti-sensitizing purposes. The latest meta-analysis also concluded that this toothpaste effectively reduces dentin sensitivity, when measured using air blast and tactile stimuli, over both short- (<2 weeks) and long-term timeframes (>2 weeks), when compared to negative controls [18].

To evaluate the effectiveness of anti-sensitivity toothpaste, appropriate study periods must be employed. A follow-up period of 3 days, 7 days, or 2 weeks was recommended for instant effect evaluation, while a 4-, 6-, 8-, or 12-week timeframe was recommended for long-lasting effect evaluations according to Bae et al. (2015) [19]. In a previous study on the effectiveness of the control toothpaste, the toothpaste was clinically validated to reduce dentin hypersensitivity within two weeks [20,21,22,23,24] and maintain its efficacy at four weeks [20,22,25]. Thus, evaluation points at 2 and 4 weeks were chosen for this study. While a longer follow-up period (>4 weeks) may more confidently assess long-term effects, it also possesses a significant confounding factor, as the dentinal tubules can naturally become occluded with calcified material over time [26], leading to a reduction in sensitivity that is not attributable to the test toothpaste. For future studies, we recommend including a negative control group to better account for this natural healing process and further validate the treatment’s efficacy.

In this study, both tested formulations led to significant reductions in VAS scores at both 2 and 4 weeks, confirming their effectiveness in alleviating dentin hypersensitivity. This result is consistent with findings from the previous study of S2 toothpaste (herbal toothpaste with potassium nitrate), which reported that VAS scores from the air blast test decreased from 6.20 ± 2.36 to 4.33 ± 2.21 at 2 weeks and 2.73 ± 2.33 at 4 weeks [16]. At the 2-week mark, the S3 group (potassium nitrate toothpaste) demonstrated the greatest reduction in sensitivity, with the lowest median VAS score being 4. However, this difference reached statistical significance only when compared with the S1 group (herbal toothpaste without potassium nitrate), suggesting that the early desensitizing effect of the herbal formulation was mainly attributable to the synthetic potassium salt content. The concentration or bioavailability of potassium salts in the herbal formulation may be insufficient to produce an immediate effect comparable to that of synthetic potassium nitrate. The superior performance of the S2 group (herbal toothpaste combined with potassium nitrate) compared to that of the S1 group (herbal toothpaste) supports this hypothesis. Additionally, the fluoride content in the S3 toothpaste may have contributed to its early efficacy by promoting the occlusion of dentinal tubules—a well-documented mechanism for reducing sensitivity [22].

By the 4-week assessment, the S1 and S3 groups demonstrated comparable efficacy, suggesting that the herbal toothpaste may require a longer duration to achieve its full effect. Interestingly, the gap between the VAS scores of the S1 (3.11 ± 1.09) and S3 groups (4.37 ± 2.84) in the air blast test was quite high, albeit not statistically different. The data also show that those in the S3 group had around 22% of teeth with a VAS ≥ 7, resulting in a high mean value. Importantly, the S2 group continued to exhibit the greatest reduction in VAS scores, with a median score of 2, and remained significantly more effective than the S3 group. This superior performance of S2 may be attributed to its enhanced dentinal tubule occlusion properties. An in vitro study reported that around half of the dentinal tubules were occluded after brushing the dentin disc with a conventional potassium nitrate toothpaste, while an herbal potassium nitrate toothpaste occluded most of them [15]. Further study is needed to explain the real mechanisms and the difference between synthetic and herbal potassium nitrate toothpaste.

Despite these improvements, only a small proportion of teeth (15 out of 115 for the tactile test and 3 out of 95 for the air blast test) achieved a VAS score of zero at 2 weeks. This result underscores the limitations of desensitizing toothpastes, which typically alleviate but do not completely resolve symptoms. This observation aligns with previous studies suggesting that such products are best used as adjunctive rather than stand-alone treatments for dentin hypersensitivity [19].

This study utilized the Visual Analog Scale (VAS) score to determine the severity of dentinal hypersensitivity. However, many studies have conducted similar experiments using the Schiff cold air sensitivity scale (SCASS): a scale ranging from 0 to 3, with each number corresponding to a specific patient response [27]. The SCASS was found to be fairly effective (Area under the Receiver Operating Characteristic curve (AUC) = 0.729; 95% CI: 0.670–0.787) at detecting major changes in tooth sensitivity with moderate sensitivity (75.7%) and very high specificity (91%). The VAS score also demonstrates comparable effectiveness (AUC = 0.747; 95% CI: 0.690–0.805) with slightly higher sensitivity (80.6%) but lower specificity (61.1%) [28]. However, the VAS score may offer an advantage in detecting minor improvements. For instance, if a patient has a tolerable level of sensitivity at the initial measurement (SCASS = 1) and begins to feel less sensitive after receiving the intervention but is not completely free from the sensation, the SCASS score would likely remain unchanged, while the VAS score would decrease. Therefore, future studies should be designed to collect both parameters to cover all aspects of sensitivity change.

To ensure consistency and minimize selection bias, only teeth with an initial VAS score ≥ 4 were included in this study, following the methodology of Pradeep et al. (2010) [23]. This criterion was chosen for two reasons: first, teeth with lower VAS scores have minimal potential for further improvement in sensitivity; second, patients with teeth exhibiting low VAS scores typically do not perceive sensitivity as a significant problem and are, therefore, not the primary target group for anti-sensitivity toothpaste.

However, researchers faced several problems when using the inclusion criterion of a VAS score of ≥4. First, VAS scores of 4 to 10 are quite wide, making it very difficult to achieve comparable initial VAS score levels for all groups. Even utilizing block randomization could not guarantee an even distribution, leading to an accumulation of teeth with lower or higher initial VAS scores within a single study group. To prevent this issue in future studies, limiting the range of included scores (e.g., to VAS scores of 7 to 10 only) or dividing subjects into subgroups based on their initial VAS scores for stratified statistical analysis could be considered. Secondly, an imbalance in sample size between groups was observed, partly because some teeth met the VAS score criterion in only one of the sensitivity tests (e.g., air blast but not tactile or vice versa). The inclusion of multiple teeth (one or two) per participant may have also contributed to this inter-group imbalance. In the present study, the S3 group appeared to be most affected by these issues. While adjusting the inclusion criteria, such as allowing only one tooth per patient or requiring a VAS score of ≥4 for both air blast and tactile tests, might reduce these imbalances, it would likely pose significant challenges in subject recruitment.

No adverse effects were reported among participants in both herbal toothpaste groups (S1 and S2). This finding is consistent with the existing literature, which reports good safety for both Java Tea and Little Ironweed. Java Tea is traditionally used for its anti-inflammatory [29], antioxidant [29], and antihypertensive properties [30]. Clinical studies have shown no adverse effects after consuming an aqueous extract containing 3.2–3.6 g of dried leaves daily for 14 days [31]. Little Ironweed is similarly recognized for its anti-inflammatory [32], anti-pyretic [33], antimalarial [34], antidiabetic [35], and smoking cessation properties [36,37,38,39], with three relevant studies reporting no serious adverse effects during a use period of up to 12 weeks [37,38,39]. In contrast, one participant in the control group reported a burning sensation and withdrew from the study, highlighting a possible drawback of conventional formulations and reinforcing the value of herbal alternatives.

## 5. Conclusions

This study demonstrated that an herbal toothpaste containing Java Tea and Little Ironweed alone is effective in reducing dentin hypersensitivity, with efficacy comparable to conventional potassium nitrate toothpaste at the 4-week period, but that it is less effective compared to the formula mixed with potassium nitrate. The addition of potassium nitrate significantly enhanced the toothpaste’s desensitizing effects, indicating the possibility of a synergistic effect. However, further analysis with an increased sample size and longer follow-up period may be needed to determine the true effectiveness of the herbal toothpaste used in this study. Additionally, comparative studies with other toothpastes exhibiting dentinal tubule-occluding properties would be beneficial to gain a more comprehensive understanding of this herbal formulation’s mechanism and efficacy.

## Figures and Tables

**Table 1:** Composition of the experimental toothpaste.

Toothpaste	Active Ingredient	Full Scientific Species
S1 toothpaste	Clinacanthus nutans	*Clinacanthus nutans* (Burm.f.) Lindau
	Orange Jessamine	*Murraya Exotica L*
	Little Ironweed	*Vernonia cinerea*
	Java Tea	*Orthosiphon aristatus*
	Hydrocotyle	*Centella asiatica (L.)* Urb
	Toothbrush Tree	*Streblus asper* Lour.
	Mangosteen	*Garcinia mangostana* Linn.
S2 toothpaste	Clinacanthus nutans	*Clinacanthus nutans* (Burm.f.) Lindau
	Orange Jessamine	*Murraya Exotica L*
	Little Ironweed	*Vernonia cinerea*
	Java Tea	*Orthosiphon aristatus*
	Hydrocotyle	*Centella asiatica (L.)* Urb
	Toothbrush Tree	*Streblus asper* Lour.
	Mangosteen	*Garcinia mangostana* Linn.
	0.7% Potassium Nitrate	
S3 toothpaste	5% Potassium Nitrate	
	0.76% Sodium Monofluorophosphate	

**Table 2:** Distribution of VAS scores for the tactile tests at the initial visit and at the 2- and 4-week follow-ups.

Timeline	VAS Score	Initial		2 Weeks		4 Weeks	
		*n*	%	*n*	%	*n*	%
S1 Group (*n* = 47)	7–10	27	57.45	8	17.02	0	0
	4–6	20	42.55	32	68.09	19	40.43
	1–3	0	0	5	10.64	26	55.32
	0	0	0	2	4.26	2	4.26
	Median	7		6		3	
S2 Group (*n* = 46)	7–10	21	46.65	4	8.70	2	4.35
	4–6	25	54.35	30	65.22	8	17.39
	1–3	0	0	7	15.22	17	36.96
	0	0	0	5	10.87	19	41.40
	Median	6		5		2	
S3 Group (*n* = 22)	7–10	12	54.55	4	18.18	1	4.55
	4–6	10	45.45	8	36.36	9	40.91
	1–3	0	0	5	22.73	6	27.27
	0	0	0	5	22.73	6	27.27
	Median	7		4		3	

VAS: Visual Analog Scale.

**Table 3:** Distribution of VAS scores for the air blast tests at the initial visit and at the 2- and 4-week follow-ups.

Timeline	VAS Score	Initial		2 Weeks		4 Weeks	
		*n*	%	*n*	%	*n*	%
S1 Group (*n* = 38)	7–10	28	73.68	11	28.95	0	0
	4–6	10	26.32	21	55.26	10	40.43
	1–3	0	0	4	10.53	26	55.32
	0	0	0	2	5.26	2	4.26
	Median	7		6		3	
S2 Group (*n* = 30)	7–10	22	73.33	0	0	0	0
	4–6	8	26.67	22	73.33	7	23.33
	1–3	0	0	8	26.67	13	43.33
	0	0	0	0	0	10	33.33
	Median	7.5		4		2	
S3 Group (*n* = 27)	7–10	16	59.26	5	18.52	6	22.22
	4–6	11	40.74	15	55.56	10	37.04
	1–3	0	0	6	22.22	9	33.33
	0	0	0	1	3.70	2	7.41
	Median	7		4		4	

VAS: Visual Analog Scale.

**Table 4:** Within-group comparison of the VAS scores for the tactile tests at the initial visit and at the 2- and 4-week follow-ups.

Test Group	1st Timeline	2nd Timeline	VAS Score of the 1st Timeline	VAS Score of the 2nd Timeline	*p*-Value *	*p*-Value **
S1 Group (*n* = 47)	Initial	2 Weeks	6.89 ± 0.98	5.38 ± 1.69	<0.001 ***	<0.001 ***
	Initial	4 Weeks	6.89 ± 0.98	3.21 ± 1.08		<0.001***
	2 Weeks	4 Weeks	5.38 ± 1.69	3.21 ± 1.08		<0.001***
S2 Group (*n* = 46)	Initial	2 Weeks	6.65 ± 1.52	4.20 ± 2.03	<0.001 ***	0.001 ***
	Initial	4 Weeks	6.65 ± 1.52	1.91 ± 2.07		<0.001 ***
	2 Weeks	4 Weeks	4.20 ± 2.03	1.91 ± 2.07		<0.001 ***
S3 Group (*n* = 22)	Initial	2 Weeks	6.82 ± 1.99	3.59 ± 2.59	<0.001 ***	0.002 ***
	Initial	4 Weeks	6.82 ± 1.99	2.95 ± 2.42		<0.001 ***
	2 Weeks	4 Weeks	3.59 ± 2.59	2.95 ± 2.42		0.366

VAS: Visual Analog Scale, * Friedman test, ** Post hoc analysis with a Bonferroni-corrected pairwise Wilcoxon signed-rank test, *** *p*-value < 0.05 indicates a statistical difference.

**Table 5:** Within-group comparison of the VAS scores for the air blast tests at the initial visit and at the 2- and 4-week follow-ups.

Test group	1st Timeline	2nd Timeline	VAS Score of the 1st Timeline	VAS Score of the 2nd Timeline	*p*-Value *	*p*-Value **
S1 Group (*n* = 38)	Initial	2 Weeks	7.39 ± 1.15	5.39 ± 1.85	<0.001 ***	0.001 ***
	Initial	4 Weeks	7.39 ± 1.15	3.11 ± 1.09		<0.001 ***
	2 Weeks	4 Weeks	5.39 ± 1.85	3.11 ± 1.09		<0.001 ***
S2 Group (*n* = 30)	Initial	2 Weeks	7.53 ± 1.31	4.13 ± 1.11	<0.001 ***	<0.001 ***
	Initial	4 Weeks	7.53 ± 1.31	2.10 ± 1.71		<0.001 ***
	2 Weeks	4 Weeks	4.13 ± 1.11	2.10 ± 1.71		0.002 ***
S3 Group (*n* = 27)	Initial	2 Weeks	6.89 ± 2.12	4.52 ± 1.97	<0.001 ***	0.005 ***
	Initial	4 Weeks	6.89 ± 2.12	4.37 ± 2.84		<0.001 ***
	2 Weeks	4 Weeks	4.52 ± 1.97	4.37 ± 2.84		0.341

VAS: Visual Analog Scale, * Friedman test, ** Post hoc analysis with a Bonferroni-corrected pairwise Wilcoxon signed-rank test, *** *p*-value < 0.05 indicates a statistical difference.

**Table 6:** Between-group comparison of the VAS scores for the tactile tests at the initial visit and at the 2- and 4-week follow-ups.

Timeline	1st Test Group	2nd Test Group	VAS Score of the 1st Test Group	VAS Score of the 2nd Test Group	*p*-Value *	*p*-Value **
Initial	S1 Group (*n* = 47)	S2 Group (*n* = 46)	6.89 ± 0.98	6.65 ± 1.52	0.392	None
	S1 Group (*n* = 47)	S3 Group (*n* = 22)	6.89 ± 0.98	6.82 ± 1.99		
	S2 Group (*n* = 46)	S3 Group (*n* = 22)	6.65 ± 1.52	6.82 ± 1.99		
2 Weeks	S1 Group (*n* = 47)	S2 Group (*n* = 46)	5.38 ± 1.69	4.20 ± 2.03	0.001 ***	0.001 ***
	S1 Group (*n* = 47)	S3 Group (*n* = 22)	5.38 ± 1.69	3.59 ± 2.59		0.002 ***
	S2 Group (*n* = 46)	S3 Group (*n* = 22)	4.20 ± 2.03	3.59 ± 2.59		0.684
4 Weeks	S1 Group (*n* = 47)	S2 Group (*n* = 46)	3.21 ± 1.08	1.91 ± 2.07	0.001 ***	<0.001 ***
	S1 Group (*n* = 47)	S3 Group (*n* = 22)	3.21 ± 1.08	2.95 ± 2.42		0.456
	S2 Group (*n* = 46)	S3 Group (*n* = 22)	1.91 ± 2.07	2.95 ± 2.42		0.035 ***

VAS: Visual Analog Scale, * Kruskal–Wallis test, ** Post hoc analysis with Dunn’s test and a Bonferroni correction, *** *p*-value < 0.05 indicates a statistical difference.

**Table 7:** Between-group comparison of the VAS scores for the air blast tests at the initial visit and at the 2- and 4-week follow-ups.

Timeline	1st Test Group	2nd Test Group	VAS Score of the 1st Test Group	VAS Score of the 2nd Test Group	*p*-Value *	*p*-Value **
Initial	S1 Group (*n* = 38)	S2 Group (*n* = 30)	7.39 ± 1.15	7.53 ± 1.31	0.306	None
	S1 Group (*n* = 38)	S3 Group (*n* = 27)	7.39 ± 1.15	6.89 ± 2.12		
	S2 Group (*n* = 30)	S3 Group (*n* = 27)	7.53 ± 1.31	6.89 ± 2.12		
2 Weeks	S1 Group (*n* = 38)	S2 Group (*n* = 30)	5.39 ± 1.85	4.13 ± 1.11	0.001 ***	0.001 ***
	S1 Group (*n* = 38)	S3 Group (*n* = 27)	5.39 ± 1.85	4.52 ± 1.97		0.018 ***
	S2 Group (*n* = 30)	S3 Group (*n* = 27)	4.13 ± 1.11	4.52 ± 1.97		0.287
4 Weeks	S1 Group (*n* = 38)	S2 Group (*n* = 30)	3.11 ± 1.09	2.10 ± 1.71	0.001 ***	0.030 ***
	S1 Group (*n* = 38)	S3 Group (*n* = 27)	3.11 ± 1.09	4.37 ± 2.84		0.117
	S2 Group (*n* = 30)	S3 Group (*n* = 27)	2.10 ± 1.71	4.37 ± 2.84		<0.001 ***

VAS: Visual Analog Scale, * Kruskal–Wallis test, ** Post hoc analysis with Dunn’s test and a Bonferroni correction, *** *p*-value < 0.05 indicates a statistical difference.

## Data Availability

The raw data supporting the conclusions of this article will be made available by the authors on request.

## Abbreviations

The following abbreviations are used in this manuscript:

VAS	Visual Analog Scale
SCASS	Schiff cold air sensitivity scale
AUC	Area under the Receiver Operating Characteristic curve
SEM	Scanning electron microscopy
